# *mpt64* mutations in *Mycobacterium tuberculosis* with negative MPT64 antigen assay results from a tertiary hospital in Southeastern China

**DOI:** 10.3389/fmed.2025.1531853

**Published:** 2025-02-26

**Authors:** Xinling Pan, Sujuan Zhou, Lulu Jin, Songjun Ji, Xingxing Lou, Bin Lu, Jin Zhao

**Affiliations:** ^1^Department of Biomedical Sciences Laboratory, Affiliated Dongyang Hospital of Wenzhou Medical University, Dongyang, Zhejiang, China; ^2^School of Public Health, Fudan University, Shanghai, China; ^3^Department of Clinical Laboratory, Affiliated Dongyang Hospital of Wenzhou Medical University, Dongyang, Zhejiang, China; ^4^Department of Infectious Diseases, Affiliated Dongyang Hospital of Wenzhou Medical University, Dongyang, Zhejiang, China

**Keywords:** *Mycobacterium tuberculosis*, MPT64, gene mutation, false negative, clinical features

## Abstract

**Background:**

MPT64 protein is an effective marker for detecting *Mycobacterium tuberculosis* (MTB) in liquid culture and clinical tissue samples. However, some MTB clinical isolates test negative for this antigen because of varied mutation types across different regions.

**Methods:**

DNA samples of MPT64 antigen assay-negative MTB strains were collected from a tertiary hospital from January 2016 to January 2024, and *mpt64* gene mutations were detected by sequencing. Clinical records of patients with negative MPT64 antigen results were collected and compared with those of patients with positive results. The global distribution of *mpt64* gene mutations was analyzed using MTB genome sequences from the National Center for Biotechnology Information (NCBI) database.

**Results:**

Among 821 mycobacterial specimens with negative MPT64 antigen assay results, 77 MTB strains were collected from 73 patients. Compared with MPT64-positive patients (*n* = 301), a higher percentage of MPT64-negative patients had a history of anti-tuberculosis therapy (*n* = 7, 11.1%; *P* = 0.01). Moreover, MPT64-negative patients demonstrated a lower percentage of positive Gene Xpert results than MPT64-positive patients (73.8% vs 95.1%, *P* < 0.001). Several gene mutations were detected in the MPT64-negative MTB strains, including 63 bp deletion, single nucleotide mutations, and *IS6110* insertion. Among 7,324 MTB genomes from the NCBI database, 87 strains had mutations in the *mpt64* gene sequence, with four common mutation sites causing single amino acid changes, including G34A (8.0%), A103G (27.6%), T128A (9.2%), and C477A (24.1%).

**Conclusion:**

A negative MPT64 antigen result in MTB cultures can be attributed to mutations in the *mpt64* gene, and infections caused by these strains are more likely to be misdiagnosed.

## Introduction

Tuberculosis remains a major public health concern worldwide, although its prevalence and incidence have gradually decreased over the years. According to the Global Tuberculosis Report of the World Health Organization, over 10.3 million individuals developed tuberculosis in 2022 (1). Disease transmission can be prevented through early molecular diagnosis and treatment using a combination of anti-tuberculous agents.

Patients with tuberculosis are screened using several markers specific to *Mycobacterium tuberculosis* (MTB) (2). MPT64, a soluble protein encoded by a gene located in the region of difference (RD) 2, is expressed in MTB isolates but not in the live attenuated *Mycobacterium bovis* (Bacillus Calmette-Guérin) vaccine strain (3). Therefore, the positive MPT64 expression in laboratory cultures and clinical samples provides direct evidence of MTB infection (4, 5). In general, MPT64 is detected via an immunochromatographic test using specific antibodies. Commonly used commercial kits include Capilia TB and SD Bioline TB Ag MPT64. While MPT64 detection in tissue specimens is superior to Gene Xpert assay and mycobacterial cultures for diagnosing extrapulmonary tuberculosis (6, 7), its diagnostic performance is heterogeneous across other specimens.

Nonetheless, inconsistencies have been observed between the MPT64 and nucleic acid-based assays for MTB infections (8, 9). The negative results are largely caused by *mpt64* gene mutations, as supported by a few studies (4, 9–12). For negative MPT64 results, further species identification usingmolecular techniques is necessary to confirm whether the strainbelongs to non-tuberculous mycobacteria (NTM). Certain MTB lineages appear to have a higher prevalence of *mpt64* mutations; however, mutation types vary across different regions (9, 12, 13). Furthermore, whether clinical features vary across patients with infections by MTB strains with negative MPT64 detection remains unknown.

## Materials and methods

### Ethical approval statement

This study was conducted per the World Medical Association Declaration of Helsinki. Verbal informed consent was obtained via telephone call upon identifying the existence of MTB strain. The study did not involve patients younger than 18 years. It was approved by the Ethics Committee and Institutional Review Board of Dongyang People’s Hospital (2023-YX-319).

### Sample collection from clinical culture specimens

First, mycobacterial culture was obtained by positive results in the BACTEC MGIT960 System (BD, United States) and confirmed by positive acid-fast test. Second, the MPT64 antigen assay was conducted in the culture supernatants using a kit (Genesis Corporation, Hangzhou, China) according to the manufacturer’s instructions. Finally, species were identified by *hsp65* amplification and sequencing, as described in our previous study (14). The heated and lysed supernatants of MPT64-negative strains were collected from patients with tuberculosis admitted to our hospital between January 2016 and January 2024 and stored at −*80*°C until further analysis.

### Retrieval of clinical data to analyze the clinical features

The clinical data of patients with positive MPT64 assay results from 2019 were retrospectively extracted from medical records. Comorbidities were identified based on physical, laboratory, and radiological examinations. Results of the acid-fast smear test and Gene Xpert assay (Cepheid, United States) using sputum and bronchoalveolar lavage samples, as well as TB T spot test using venous blood samples, were collected. The history of anti-tuberculosis therapy was obtained by consulting the medical history, regardless of whether the treatment was completed. Additionally, computer tomography images showing nodules, cavities, and pleural effusion were obtained from medical records.

### Molecular assays for MTB strains with negative mpt64 antigen assay results

The *mpt64* gene was amplified using primers specific for regions outside the coding sequence (12). The RD105 sequence was detected using two pairs of primers targeting the deleted type and intact type, respectively (15, 16). For samples with an intact RD105, the pks15/1 region was amplified and sequenced. The Beijing family strain was defined based on the RD105 deletion (17), whereas the non-Beijing family type was further analyzed by detecting RD239 and RD711 and sequencing pks15/1 (18–20). Amplification condition and procedure have been described previously (14). The amplified products were analyzed by electrophoresis in 1% agarose gel. The sequence of *mpt64* was obtained by Sanger sequencing (GENEWIZ, China). The primer sequences are listed in Supplementary Table 1.

### Genome sequence analysis for mutated *mpt64* genes

All assembled genomes of MTB were accessed from the National Center for Biotechnology Information (NCBI) genome database (by June 15, 2024) and downloaded using the NCBI Datasets command-line tool “datasets.” The *mpt64* gene sequences were extracted using the R “seqinir” and “Biostrings” packages and matched using two flanking regions (CTAGGCCAGCATCGAGTCGA and ATGAAGATCTTGATGCGCAC). Genomes without matched sequences or mutations, compared with the H37Rv-derived *mpt64* gene, were ignored. The sequences were complementally reversed and aligned using MEGA version 11.0.13 (21). The DNA sequences were translated into amino acid sequences to confirm the impact of gene mutation on the encoded protein.

### Statistical analysis

Categorical data were expressed as numbers with percentages, and differences between groups were analyzed by chi-square test. Continuous data were expressed as mean ± standard deviation, and significance was determined using independent-sample *t*-tests. All analyses were conducted using the Statistical Package for the Social Sciences Software version 27 (International Business Machines Corporation, United States).

## Results

### Clinical characteristics of patients infected with MPT64-negative MTB

A total of 821 mycobacterial strains with negative MPT64 antigen assay results were selected. After excluding 744 NTM strains, 77 MTB strains were isolated from 73 patients who were negative for the MPT64 assay. After excluding cases with missing clinical data and repeated strains from the same patients, 64 MTB strains corresponding to 64 cases were analyzed. Compared with MPT64-positive patients with tuberculosis (*n* = 8, 2.9%), MPT64-negative patients (*n* = 7, 11.1%) demonstrated a higher percentage of a history of anti-tuberculosis therapy (*P* = 0.01, Table 1). Only one MPT64-negative patient (*n* = 1, 1.6%) had chronic obstructive pulmonary disease, lower than 7.9% of MPT64-positive patients (*P* = 0.093). Moreover, MPT64-negative patients demonstrated a lower percentage of positive Gene Xpert results in the original specimens than MPT64-positive patients (73.8% vs 95.1%, *P* < 0.001).

**TABLE 1:** The clinical features for tuberculosis patients with different MPT64 antigen assay results.

Features	Values	MPT64 assay		
		Negative	Positive	*P*
Gender	Male	36 (56.3)	204 (67.8)	0.078
	Female	28 (43.7)	97 (32.2)	–
Age	Years	60.02 ± 20.44	56.5 ± 21.2	0.226
Treatment history	Yes	7 (11.1)	8 (2.9)	0.01*
	No	56 (88.9)	268 (97.1)	–
Cough	Yes	43 (76.8)	225 (80.6)	0.51
	No	13 (23.2)	54 (19.4)	–
Fever	Yes	22 (39.3)	97 (34.9)	0.531
	No	34 (60.7)	181 (65.1)	–
Hemoptysis	Yes	8 (14.3)	35 (12.6)	0.73
	No	48 (85.7)	243 (87.4)	–
Nodules in lung	Yes	39 (70.9)	182 (67.4)	0.612
	No	16 (29.1)	88 (32.6)	–
Cavity in lung	Yes	20 (36.4)	105 (38.9)	0.726
	No	35 (63.6)	165 (61.1)	–
Pleural effusion	Yes	8 (14.5)	37 (13.7)	0.869
	No	47 (85.5)	233 (86.3)	–
Bronchiectasis	Yes	4 (6.5)	13 (4.6)	0.553
	No	58 (93.5)	267 (95.4)	–
COPD	Yes	1 (1.6)	22 (7.9)	0.093
	No	61 (98.4)	258 (92.1)	–
Tuberculous pleurisy	Yes	5 (8.1)	30 (10.7)	0.533
	No	57 (91.9)	250 (89.3)	–
AFB	Positive	27 (43.5)	119 (43.3)	0.968
	Negative	35 (56.5)	156 (56.7)	–
T. spot assay	Positive	37 (97.4)	168 (96.6)	1.00*
	Negative	1 (2.6)	6 (3.4)	–
Gene Xpert	Positive	31 (73.8)	214 (95.1)	< 0.001
	Negative	11 (26.2)	11 (4.9)	–
Time to positive report	Days	49.76 ± 17.96	25.35 ± 12.96	0.274

*Fisher exact test. COPD, chronic obstructive pulmonary disease.

### Landscape of *mpt64* mutations in the MPT64-negative MTB strains

The *mpt64* sequences of all MTB strains with negative MPT64 results were amplified and sequenced. A 63 bp region was deleted in 53 strains, resulting in a truncated protein consisting of 21 amino acids, compared with the wild-type protein (Table 2). Two strains had a copy of the *IS6110* sequence in the *mpt64* gene at the 313 bp position, which introduced a stop codon leading to premature termination. Finally, one strain had a 13 bp insertion, which resulted into a longer novel protein sequence.

**TABLE 2:** The distribution of mutation types in the *mpt64* gene in *Mycobacterium tuberculosis* (MTB) strains with negative MPT64 assay.

Isolate no (%)	Mutation type	Changed nucleotides	Position in gene	Product length	Mutation outcomes	Lineages (%)
5 (8.1)	Wide type	NA	NA	228	NA	Beijing (60%)
53 (85.5)	Deletion	63	197–259	207	21 amino acids truncated	Non-Beijing (100%)
2 (3.2)	Insertion	1,370	313	135	Pretermination	Beijing (100%)
1 (1.6)	Insertion	13	635	250	22 amino acids prolonged	Beijing (100%)
1 (1.6)	Replacement	1	671	228	D224G	Beijing (100%)

### Determining MTB lineages based on region of difference

The 63 bp deletion was exclusively present in strains with the RD105 region, which corresponded to the non-Beijing family genotype. Strains with other mutation types belonged to the Beijing family genotype. Furthermore, the non-Beijing family strains had intact RD239, RD711, TbD1, and RD750 regions. The pks15/1 sequences were identical to the H37Rv-derived sequence. Based on these sequence markers, all strains with the 63 bp deletion belonged to the Euro-American lineage.

### Amino acid changes in mpt64 caused by gene polymorphisms

A total of 7,324 MTB genomes were obtained from the NCBI, of which 87 strains showed *mpt64* mutations, compared with the reference strain (Table 3). G34A (8%), A103G (27.6%), T128A (9.2%), and C477A (24.1%) were the most common sites with single amino acid changes. All strains with G34A mutations were isolated from Peru, whereas strains with other mutations were distributed in different regions (Supplementary Table 2). Furthermore, two strains had a 21 amino acid deletion because of the 63 bp deletion. Other mutated loci were distributed sporadically in MTB isolates.

**TABLE 3:** The distribution of mutation sites across the *mpt64* gene from accessible genomes of *Mycobacterium tuberculosis*.

Isolate no	Mutation type	Gene mutation (wide/mutated)	position in gene	Amino acid mutation (wide/mutated)
7	Missense	G/A	34	V/I
24	Missense	A/G	103	T/A
8	missense	T/A	128	I/N
1	synonymous	T/C	167	None
1	synonymous	G/C	241	None
1	missense	G/A	260	R/H
1	synonymous	A/G	272	None
1	synonymous	A/G	281	None
2	synonymous	C/G	306	None
1	synonymous	G/A	318	None
3	synonymous	G/A	345	None
2	synonymous	C/G	348	None
1	missense	T/G	464	L/R
21	missense	C/A	477	F/L
3	missense	C/T	480	A/D
1	synonymous	C/A	539	None
2	missense	G/A	586	G/R
3	synonymous	G/A	609	None
1	missense	G/A	634	G/D
2	deletion	63bp deletion	197–259	21aa deletion

## Discussion

The MPT64 antigen assay is routinely used to detect MTB in mycobacterial cultures and clinical samples. However, negative results caused by *mpt64* mutations can lead to misdiagnosis in the absence of other molecular assays. In practice, a cultured mycobacterial strain with a negative MPT64 assay result is considered an NTM, which requires a different drug regimen, compared with MTB (8, 22). Moreover, patients with NTM infection have not been routinely monitored in most regions because NTM rarely leads to transmission in immunocompetent individuals. Moreover, multiple antibiotic agent-containing therapy is not recommended for patients with mild symptoms (23). Thus, false-negative results in the MPT64 assay possibly resulted in MTB misdiagnosis as NTM infection, causing more susceptible individuals becoming infected by MPT64-negative patients because of a lack of therapy or resistance acquirement from an inappropriate drug regimen (24, 25). For mycobacterial strains with negative MPT64 assay results, additional time and money are warranted to conduct nucleotide acid-based assays using mycobacterial culture to identify the mycobacterial species (8). However, alternative assays (next-generation sequencing, nucleotide acid amplification, and nucleic acid probe detection) for all mycobacterial cultures are not recommended because of higher costs and the need for professional instruments and trained staff.

With the application of several molecular detection techniques in clinical laboratories for mycobacterial identification, negative MPT64 antigen assay results in MTB strains have been reported globally (8, 10–13, 26). Nevertheless, the clinical relevance of negative MPT64 antigen results has not been thoroughly investigated. In this study, a higher percentage of patients who tested negative for the MPT64 antigen received anti-tuberculosis treatment previously. This phenomenon could be partially explained by the insufficient duration of routine treatment for these patients because MPT64-negative MTB strains might have lower division activity than MPT64-positive patients (8, 27). The bacilli load influences the detection of the MPT64 antigen because the cultured MTB strains negative for the MPT64 antigen are more likely to be negative for MTB-specific nucleic acids in clinical samples, such as Gene Xpert assay (8). Moreover, the time to report tended to be longer for strains with negative MPT64 assay results, despite no significance in this study, indicating the possibility of a lower bacilli load in the specimen.

*Mpt64* mutations are the major reason for a negative MPT64 result (8, 10, 12, 13). *Mpt64* mutation types appear to have a specific association with certain lineages (12, 26). In this study, all strains carrying the 63 bp deletion in the *mpt64* gene belonged to the non-Beijing lineage, aligning with previous data suggesting that this deletion is commonly observed in L4 and L1 lineages (10, 13). The truncated protein encoded by the *mpt64* gene with a 63 bp deletion may not be recognized by the antibodies in enzyme-linked immunosorbent assay or immunochromatographic commercial kits, yielding false-negative results (10). A 63 bp deletion is predicted to drastically change the protein structure because of an alpha helix, explaining the phenomenon (13).

Single-nucleotide mutations have been reported in the *mpt64* gene. Missense mutations can alter protein structure and stability; however, the corresponding strains remained positive for the MPT64 protein assay (9, 11, 13). For example, a common mutation at 477 bp that changes phenylalanine to leucine does not affect MPT64 antigen detection (9, 11). Similar results have been reported for the missense mutation at 128 T > A, which appears specific to L5 lineage strains (9). Furthermore, a longer incubation period (8, 12) or the use of L-J culture instead of liquid culture (8) may reverse the decreased sensitivity of the MPT64 assay because of a single amino acid change. For MTB strains with negative MPT64 assay results from Australia, the *mpt64* mutation type is the deletion of two nucleotides, resulting in a frameshift in protein expression (26). Further analysis indicated that all the strains carrying this mutation belong to Lineage 4, which has been attributed to European migration and colonization (28). *IS6110* insertion is an uncommon *mpt64* mutation, and previously reported insertion sites (501 bp) (29) is different from our study (313 bp). The influence of *IS6110* on protein structure or function depends on the insertion site (30). In detail, insertion at 313 bp in this study introduced a termination codon, resulting in a shorter polypeptide of 135 amino acids.

*Mpt64* mutations are specific to certain MTB lineages or sublineages; nonetheless, whether these strains possess unique features concerning resistance phenotype and virulence remains elusive (28, 31). In the future, multi-omic investigations might elucidate the mechanism by which these strains interact with geographic populations (32–34). Moreover, the lower sensitivity of the MPT64 antigen-based assay could be overcome by combining molecular assays for different targets or adopting more convenient and economical methods for MTB diagnosis (35, 36). Furthermore, novel antibodies detecting both the mutated and wild MPT64 proteins would improve diagnostic efficiency in liquid cultures and minimize false negatives (37).

This study has several limitations. MTB strains were collected from a single center, which may not entirely represent broader genetic diversity. Strains carrying the wild-type *mpt64* gene might have yielded positive results after longer incubation in liquid culture; however, these strains were not preserved and only the nucleic acids were available. Furthermore, clinical information for some patients was missing because of retrospective data collection, particularly for outpatients, possibly introducing bias in the comparison of clinical characteristics. Finally, detailed lineage data was inaccessible for some strains because of the lower DNA quality. In the future, whole genome sequencing of mutated MTB strains would provide a more comprehensive mutational landscape.

## Conclusion

Patients with MTB infections who are negative for the MPT64 antigen are more likely to be misdiagnosed as having NTM because of lower positive rates in the Gene Xpert assay. *Mpt64* mutations, primarily the 63 bp deletion and *IS6110* insertion, were associated with negative MPT64 antigen assay results. In contrast, single nucleotide mutations in the *mpt64* gene partly contributed to the lower sensitivity of antigen assays, which may be improved by prolonged incubation. Other nucleic acid-based tests for MTB should be conducted for patients who tested negative for the MPT64 antigen but positive for mycobacterial culture.

## Data Availability

The authors acknowledge that the data presented in this study must be deposited and made publicly available in an acceptable repository, prior to publication. Frontiers cannot accept a manuscript that does not adhere to our open data policies.
