# Supplementary material for: mpt64 mutations in Mycobacterium tuberculosis with negative MPT64 antigen assay results from a tertiary hospital in Southeastern China
Source: Front Med (Lausanne). 2025 Feb 26;12:1531853. doi: 10.3389/fmed.2025.1531853 (PMC11898740; doi:10.3389/fmed.2025.1531853)
Supplement: Supplementary file 1 [file Table_1.docx]

Supplementary Table 1. The primers for amplification and sequencing used in this study.

| Genes | Forward | Reverse | Source |
| --- | --- | --- | --- |
| *HSP65* | **ATCGCCAAGGAGATCGAGCT** | AAGGTGCCGCGGATCTTGTT | Ref 14 |
| *MPT64* | TTCTTGAGCTCCCGGCCTGT | **ATCGCGGCAATCCAATCTCCC** | Ref 10 |
| RD105 intact | CGTGCACAGTTGGGTGTTTA | TTCGTTCAGGAACTCCAAGG | Ref 16 |
| RD105 deleted | CGTGCACAGTTGGGTGTTTA | CGTCGTTTTCTGCCGATACT | Ref 15 |
| Pks15/1 | **GTGTCCTCCTTTGGGATCAG** | CTGCCCAGGAAACACGAC | Ref 18 |
| RD711 | GGCCGCCCTGCTCAAGAACCT | CCTAGGCCGGCGACGAAGTG | Ref 19 |
| RD750 | GTCGGCGGTCTGCTTCGTTCC | CCTGTCGGCCGGGTGTCTTTC | Ref 19 |
| TbD1 | ACGCGGCTGGATGGTGCTGGTTG | CGCGGGCAGGGTCGTCGTGA | Ref 19 |

Notes: the bold ones were used as the sequencing primers.
