# Supplementary material for: mpt64 mutations in Mycobacterium tuberculosis with negative MPT64 antigen assay results from a tertiary hospital in Southeastern China
Source: Front Med (Lausanne). 2025 Feb 26;12:1531853. doi: 10.3389/fmed.2025.1531853 (PMC11898740; doi:10.3389/fmed.2025.1531853)
Supplement: Supplementary file 2 [file Table_2.docx]

Supplementary table 2. The distribution of mutation types in the *mpt64* gene among MTB strains from NCBI GENOMIC DATABASE.

| Accessible number | Country | Mutation position in nucleotide acid | | | | | | | | | | | | | | | | | | |  |
| --- | --- | --- | --- | --- | --- | --- | --- | --- | --- | --- | --- | --- | --- | --- | --- | --- | --- | --- | --- | --- | --- |
|  |  | 34 | 103 | 128 | 167 | 241 | 260 | 272 | 281 | 306 | 318 | 345 | 348 | 464 | 477 | 480 | 539 | 586 | 609 | 634 | 63bp |
| H37Rv (Reference) |  | G | A | T | T | G | G | A | A | C | G | G | C | T | C | C | C | G | G | G |  |
| GCF_000828995.1 | Japan | . | . | . | . | . | . | . | . | G | . | . | . | . | . | . | . | . | . | . |  |
| GCF_037076295.1 | Vietnam | . | . | . | . | . | . | . | . | . | . | . | . | . | . | . | . | . | . | A |  |
| GCF_000738445.1 | China | . | . | . | . | . | . | . | . | . | . | . | . | G | . | . | . | . | . | . |  |
| GCF_001545015.1 | China | . | . | . | . | . | . | . | . | . | . | . | . | . | . | . | . | . | . | . | Del |
| GCF_002446915.1 | Peru | . | G | . | . | . | . | . | . | . | . | . | . | . | . | . | . | . | . | . |  |
| GCF_002447055.1 | Peru | . | G | . | . | . | . | . | . | . | . | . | . | . | . | . | . | . | . | . |  |
| GCF_002447075.1 | Peru | . | G | . | . | . | . | . | . | . | . | . | . | . | . | . | . | . | . | . |  |
| GCF_002447475.1 | Peru | . | G | . | . | . | . | . | . | . | . | . | . | . | . | . | . | . | . | . |  |
| GCF_002447615.1 | Peru | . | G | . | . | . | . | . | . | . | . | . | . | . | . | . | . | . | . | . |  |
| GCF_002447655.1 | Peru | . | G | . | . | . | . | . | . | . | . | . | . | . | . | . | . | . | . | . |  |
| GCF_002447735.1 | Peru | A | . | . | . | . | . | . | . | . | . | . | . | . | . | . | . | . | . | . |  |
| GCF_002448055.1 | Peru | . | G | . | . | . | . | . | . | . | . | . | . | . | . | . | . | . | . | . |  |
| GCF_014899365.1 | Sweden | . | . | . | . | . | . | . | . | . | . | . | . | . | A | . | . | . | . | . |  |
| GCF_014899485.1 | Sweden | . | . | . | . | . | . | G | . | . | . | . | . | . | . | . | . | . | . | . |  |
| GCF_014900895.1 | India | . | . | . | . | . | . | . | . | . | . | . | . | . | A | . | . | . | . | . |  |
| GCF_021559895.1 | Benin | . | . | A | . | . | . | . | . | . | . | . | . | . | . | . | . | . | . | . |  |
| GCF_022870225.1 | Sierra Leone | . | . | A | . | . | . | . | . | . | . | . | . | . | . | . | . | . | . | . |  |
| GCF_022870245.1 | Ghana | . | . | A | . | . | . | . | . | . | . | . | . | . | . | . | . | . | . | . |  |
| GCF_022870265.1 | Ghana | . | . | A | . | . | . | . | . | . | . | . | . | . | . | . | . | . | . | . |  |
| GCF_015686915.1 | Malaysia | . | . | . | . | . | . | . | . | . | A | . | . | . | . | . | . | . | . | . |  |
| GCF_018596165.1 | Nepal | . | . | . | . | . | . | . | . | . | . | . | . | . | A | . | . | . | . | . |  |
| GCF_018596175.1 | Nepal | . | . | . | . | . | . | . | . | . | . | . | . | . | A | . | . | . | . | . |  |
| GCF_040063225.1 | Philippines | . | . | . | . | . | . | . | . | . | . | . | . | . | . | . | . | A | . | . |  |
| GCF_000651755.1 | South Africa | . | . | . | . | . | . | . | . | . | . | A | . | . | . | . | . | . | . | . |  |
| GCF_000651795.1 | South Africa | . | . | . | . | . | . | . | . | . | . | A | . | . | . | . | . | . | . | . |  |
| GCF_000652195.1 | South Africa | . | . | . | . | . | . | . | . | . | . | . | . | . | A | . | . | . | . | . |  |
| GCF_000652315.1 | South Africa | . | . | . | . | . | . | . | . | . | . | A | . | . | . | . | . | . | . | . |  |
| GCF_000652735.1 | South Africa | . | . | . | . | . | . | . | . | . | . | . | . | . | A | . | . | . | . | . |  |
| GCF_000654235.1 | India | . | . | . | . | . | . | . | . | . | . | . | . | . | A | . | . | . | . | . |  |
| GCF_000654655.1 | India | . | . | . | . | . | . | . | . | . | . | . | . | . | A | . | . | . | . | . |  |
| GCF_000654855.1 | India | . | . | . | . | . | . | . | . | . | . | . | . | . | A | . | . | . | . | . |  |
| GCF_000659005.1 | Sweden | . | . | . | . | . | . | . | . | . | . | . | . | . | A | . | . | . | . | . |  |
| GCF_000662545.1 | India | . | . | . | . | . | . | . | . | . | . | . | . | . | A | . | . | . | . | . |  |
| GCF_000662565.1 | India | . | . | . | . | . | . | . | . | . | . | . | . | . | A | . | . | . | . | . |  |
| GCF_000662585.1 | India | . | . | . | . | . | . | . | . | . | . | . | . | . | A | . | . | . | . | . |  |
| GCF_000662625.1 | India | . | . | . | . | . | . | . | . | . | . | . | . | . | A | . | . | . | . | . |  |
| GCF_000662845.1 | India | . | . | . | . | . | . | . | . | . | . | . | . | . | A | . | . | . | . | . |  |
| GCF_000662925.1 | India | . | . | . | . | . | . | . | . | . | . | . | . | . | A | . | . | . | . | . |  |
| GCF_000663025.1 | India | . | . | . | . | . | . | . | . | . | . | . | . | . | A | . | . | . | . | . |  |
| GCF_000665945.1 | Unknown | . | . | A | . | . | . | . | . | . | . | . | . | . | . | . | . | . | . | . |  |
| GCF_000666225.1 | Mali | . | . | A | . | . | . | . | . | . | . | . | . | . | . | . | . | . | . | . |  |
| GCF_000666245.1 | Mali | . | . | . | . | . | . | . | . | . | . | . | . | . | . | . | . | . | A | . |  |
| GCF_000666625.1 | Mali | . | . | A | . | . | . | . | . | . | . | . | . | . | . | . | . | . | . | . |  |
| GCF_000666825.1 | Mali | . | . | . | C | . | . | . | . | . | . | . | . | . | . | . | . | . | . | . |  |
| GCF_000667305.1 | Mali | . | . | . | . | . | . | . | . | . | . | . | . | . | . | . | . | . | A | . |  |
| GCF_000667345.1 | Mali | . | . | . | . | . | . | . | . | . | . | . | . | . | . | . | . | . | A | . |  |
| GCF_000668115.1 | China | . | . | . | . | . | . | . | . | . | . | . | . | . | . | . | . | A | . | . |  |
| GCF_000669095.1 | Uganda | . | G | . | . | . | . | . | . | . | . | . | . | . | . | . | . | . | . | . |  |
| GCF_000669695.1 | China | . | . | . | . | . | . | . | . | G | . | . | . | . | . | . | . | . | . | . |  |
| GCF_000669715.1 | Unknown | . | . | . | . | . | . | . | . | . | . | . | G | . | . | . | . | . | . | . |  |
| GCF_000672855.1 | South Africa | . | . | . | . | . | . | . | . | . | . | . | . | . | A | . | . | . | . | . |  |
| GCF_000673135.1 | India | . | . | . | . | . | . | . | . | . | . | . | . | . | A | . | . | . | . | . |  |
| GCF_000673215.1 | Unknown | . | . | . | . | . | . | . | . | . | . | . | . | . | A | . | . | . | . | . |  |
| GCF_000678335.1 | Sweden | . | . | . | . | . | . | . | . | . | . | . | . | . | . | T | . | . | . | . |  |
| GCF_000678595.1 | Sweden | . | G | . | . | . | . | . | . | . | . | . | . | . | . | . | . | . | . | . |  |
| GCF_000679195.1 | Sweden | . | . | . | . | . | . | . | . | . | . | . | . | . | . | T | . | . | . | . |  |
| GCF_000679955.1 | Belarus | . | . | . | . | . | . | . | . | . | . | . | . | . | . | . | A | . | . | . |  |
| GCF_905183075.1 | France | . | . | A | . | . | . | . | . | . | . | . | . | . | . | . | . | . | . | . |  |
| GCF_001844825.1 | Peru | A | . | . | . | . | . | . | . | . | . | . | . | . | . | . | . | . | . | . |  |
| GCF_001850135.1 | Peru | . | G | . | . | . | . | . | . | . | . | . | . | . | . | . | . | . | . | . |  |
| GCF_001842095.1 | Peru | . | G | . | . | . | . | . | . | . | . | . | . | . | . | . | . | . | . | . |  |
| GCF_001842045.1 | Peru | . | G | . | . | . | . | . | . | . | . | . | . | . | . | . | . | . | . | . |  |
| GCF_001848805.1 | Peru | . | G | . | . | . | . | . | . | . | . | . | . | . | . | . | . | . | . | . |  |
| GCF_001848505.1 | Peru | . | G | . | . | . | . | . | . | . | . | . | . | . | . | . | . | . | . | . |  |
| GCF_001851575.1 | Peru | A | . | . | . | . | . | . | . | . | . | . | . | . | . | . | . | . | . | . |  |
| GCF_001851205.1 | Peru | . | G | . | . | . | . | . | . | . | . | . | . | . | . | . | . | . | . | . |  |
| GCF_001850465.1 | Peru | . | G | . | . | . | . | . | . | . | . | . | . | . | . | . | . | . | . | . |  |
| GCF_001853265.1 | Peru | . | G | . | . | . | . | . | . | . | . | . | . | . | . | . | . | . | . | . |  |
| GCF_001943385.1 | Peru | . | G | . | . | . | . | . | . | . | . | . | . | . | . | . | . | . | . | . |  |
| GCF_001944525.1 | Peru | A | . | . | . | . | . | . | . | . | . | . | . | . | . | . | . | . | . | . |  |
| GCF_001948155.1 | Peru | . | G | . | . | . | . | . | . | . | . | . | . | . | . | . | . | . | . | . |  |
| GCF_001948215.1 | Peru | A | . | . | . | . | . | . | . | . | . | . | . | . | . | . | . | . | . | . |  |
| GCF_001945715.1 | Peru | . | G | . | . | . | . | . | . | . | . | . | . | . | . | . | . | . | . | . |  |
| GCF_001945935.1 | Peru | A | . | . | . | . | . | . | . | . | . | . | . | . | . | . | . | . | . | . |  |
| GCF_002130695.1 | Peru | . | G | . | . | . | . | . | . | . | . | . | . | . | . | . | . | . | . | . |  |
| GCF_002131245.1 | Peru | . | . | . | . | . | A | . | . | . | . | . | . | . | . | . | . | . | . | . |  |
| GCF_000008585.1 | Unknown | . | . | . | . | . | . | . | . | . | . | . | G | . | . | . | . | . | . | . |  |
| GCF_000389905.1 | Unknown | . | . | . | . | . | . | . | . | . | . | . | . | . | . | . | . | . | . | . |  |
| GCF_029867225.1 | Peru | . | G | . | . | . | . | . | . | . | . | . | . | . | . | . | . | . | . | . |  |
| GCF_030566675.1 | China | . | . | . | . | . | . | . | . | . | . | . | . | . | . | T | . | . | . | . |  |
| GCF_030571635.1 | China | . | . | . | . | C | . | . | . | . | . | . | . | . | . | . | . | . | . | . |  |
| GCF_000660045.1 | Unknown | . | . | . | . | . | . | . | . | . | . | . | . | . | . | . | . | . | . | . | Del |
| GCF_000661425.1 | South Africa | . | . | . | . | . | . | . | . | . | . | . | . | . | A | . | . | . | . | . |  |
| GCF_002976355.1 | Canada | . | . | . | . | . | . | . | G | . | . | . | . | . | . | . | . | . | . | . |  |
| GCF_004108185.1 | Peru | . | G | . | . | . | . | . | . | . | . | . | . | . | . | . | . | . | . | . |  |
| GCF_004110565.1 | Peru | A | . | . | . | . | . | . | . | . | . | . | . | . | . | . | . | . | . | . |  |
| GCF_004110645.1 | Peru | . | G | . | . | . | . | . | . | . | . | . | . | . | . | . | . | . | . | . |  |
